# Iron Deficiency in Acute Decompensated Heart Failure

**DOI:** 10.3390/jcm8101569

**Published:** 2019-10-01

**Authors:** Anna Beale, David Carballo, Jerome Stirnemann, Nicolas Garin, Thomas Agoritsas, Jacques Serratrice, David Kaye, Philippe Meyer, Sebastian Carballo

**Affiliations:** 1 Baker Heart & Diabetes Institute, 70 Commercial Rd, Melbourne (VIC) 3004, Australia; David.Kaye@baker.edu.au; 2 Cardiology Unit, The Alfred Hospital, 55 Commercial Rd, Melbourne (VIC) 3004, Australia; 3 Monash University, Wellington Rd, Clayton, Melbourne (VIC) 3800, Australia; 4 Service of Cardiology, University Hospitals of Geneva, Rue Gabrielle-Perret-Gentil 4, 1205 Geneva, Switzerland; David.Carballo@hcuge.ch (D.C.); philippe.meyer@hcuge.ch (P.M.); 5 Service of General Internal Medicine, University Hospitals of Geneva, Rue Gabrielle-Perret-Gentil 4, 1205 Geneva, Switzerland; Jerome.Stirnemann@hcuge.ch (J.S.); nicolas.garin@hcuge.ch (N.G.); thomas.agoritsas@hcuge.ch (T.A.); jacques.serratrice@hcuge.ch (J.S.); Sebastian.Carballo@hcuge.ch (S.C.)

**Keywords:** iron deficiency, heart failure, heart failure with preserved ejection fraction, length of stay

## Abstract

The aim of this study was to characterize iron deficiency (ID) in acutely decompensated heart failure (ADHF) and identify whether ID is associated with dyspnea class, length of stay (LOS), biomarker levels, and echocardiographic indices of diastolic function in patients with reduced ejection fraction (HFrEF) and with preserved ejection fraction (HFpEF). Consecutive patients admitted with ADHF at a single tertiary center were included. Demographic information, pathology investigations, and metrics regarding hospital stay and readmission were recorded. Patients were classified as having ‘absolute’ ID if they had a ferritin level <100 ng/mL; or ‘functional’ ID if they had a ferritin 100–200 ng/mL and a transferrin saturation <20%. Of 503 patients that were recruited, 270 (55%) had HFpEF, 160 (33%) had HFREF, and 57 (12%) had heart failure with mid-range ejection fraction. ID was present in 54% of patients with HFrEF and 56% of patients with HFpEF. In the HFpEF group, ID was associated with a LOS of 11 ± 7.7 vs. 9 ± 6 days in iron replete patients, *p* = 0.036, and remained an independent predictor of increased LOS in a multivariate linear regression incorporating comorbidities, age, and ID status. This study corroborates a high prevalence of ID in both HFrEF and HFpEF, and further shows that in patients with HFpEF there is a prolongation of LOS not seen in HFrEF which may indicate a more prominent role for ID in HFpEF.

## 1. Introduction

Iron deficiency (ID) is a common comorbidity in patients with heart failure [1]. Prevalence is similar in patients with heart failure with reduced ejection fraction (HFrEF), heart failure with mid-range ejection fraction (HFmrEF), and heart failure with preserved ejection fraction (HFpEF), at 50%, 61%, and 64% respectively [2]. In these patients, ID can arise not only from low whole body ferritin stores, termed absolute ID and which is multifactorial including intestinal dysfunction with reduced absorption due to reduced gastric emptying and mucosal edema, along with reduced consumption of iron rich foods due to anorexia, and increased blood loss due to anticoagulants and gastric ulceration [3,4]; but also from a rise in the regulatory protein hepcidin, which prevents ferritin release from enterocytes and macrophages in response to an inflammatory state, causing functional ID [5].

Mounting evidence supports a key role of ID in heart failure outcomes. In patients with HFrEF, there is comprehensive data illustrating that ID is related to worse dyspnea class, higher biomarker levels, lower exercise capacity, lower quality of life, and greater risk of death and heart transplantation [1,6,7,8]. Treatment of iron deficiency, in keeping with these findings, has focused on patients with HFrEF. Intravenous ferric carboxymaltose administration is associated with improvement of quality of life, reduced hospitalizations, increased exercise tolerance, and maximal oxygen consumption (VO_2_ max) in HFrEF [9,10,11,12]. Of note, this is not the case with oral iron repletion [13]. 

There is less data supporting a role of ID in HFpEF pathogenesis and prognosis. ID is nevertheless associated with lower VO_2_ max in all three phenotypes of heart failure [2]. This lends weight to previous findings suggesting that ID is related to functional outcomes in advanced HFpEF [14]. Furthermore, absolute ID has been associated with increased risk of 30-day readmission in a population of patients of whom HFpEF represented just over half [15]. However, this association has not been investigated for HFpEF and HFrEF separately. A role for ID in the pathogenesis and severity of HFpEF could indicate potential therapeutic benefit of ferric carboxymaltose in this population for whom pharmacological options are lacking [16], and which is currently under investigation in the FAIR-HFpEF trial (NCT03074591). Furthermore, whilst the prevalence of iron deficiency in acute decompensated heart failure is demonstrably high [17], data is lacking for the significance of ID in this cohort. 

In this study we sought to identify whether there is an association between ID and clinical entities such as dyspnea class, length of stay, biomarker levels, and echocardiographic indices of diastolic function in patients with acute decompensated heart failure (ADHF) in both HFrEF and HFpEF.

## 2. Methods

### 2.1. Study Design and Population

Patients were recruited in a prospective registry on acute heart failure between November 2014 and August 2017 at the University Hospitals of Geneva, ClinicalTrials.gov NCT02444416. The cohort comprised consecutive patients admitted to the departments of General Internal Medicine with ADHF, according to the European Society of Cardiology definition; exhibiting symptoms of heart failure including dyspnea, ankle swelling, and fatigue, accompanied by signs of heart failure such as elevated jugular venous pressure, pulmonary crackles, and peripheral edema due to structural or functional cardiac abnormality [18]. In addition, patients were required to have elevated brain natriuretic peptide (BNP) levels >100 ng/L, or pro-BNP levels >300 ng/L to be included in the study. This study complies with the Declaration of Helsinki, and was approved by the local medical ethics committee, and all cohort participants signed an informed consent form.

### 2.2. Study and Laboratory Measurements

Demographic information, including age, sex, weight, smoking status, and a full medical history was recorded. Treatment history at presentation and medical therapy through the course of admission was noted, focusing on heart failure therapies. Clinical status at presentation, including New York Heart Association (NYHA) dyspnea class, was noted. Routine investigations as part of the admission with ADHF were registered for each patient, including full blood examination, urea and electrolytes, C-reactive protein (CRP), iron studies, liver function tests, troponin levels, electrocardiography, and echocardiography. BNP levels were recorded until March 2015, followed by recording of N-terminal prohormone of BNP (NT-proBNP) afterwards. Echocardiography data were analyzed by staff cardiologists.

Participants were classified as having HFpEF if they had a left ventricular ejection fraction (LVEF) ≥50%; HFrEF if they had a LVEF <40%; and heart failure with mid range ejection fraction (HFmrEF) if they had a LVEF ≥40% and <50%. Iron deficiency was defined as either absolute iron deficiency, with a ferritin level <100 ng/mL; or functional iron deficiency, with a ferritin 100–200 ng/mL accompanied by a transferrin saturation <20%; in accordance with current consensus in the literature for the diagnosis of iron deficiency in heart failure.

### 2.3. Statistical Analysis

Categorical variables are presented as numbers with percentages of patients; continuous variables are presented as means and standard deviations if approximately normally distributed, or with medians with interquartile ranges otherwise. Differences in LVEF and age were compared using the independent samples t-test, whilst the difference in gender, comorbidities, and NYHA class were compared with the chi-square test for independence. The differences in iron status, biomarkers including CRP and BNP/NT-proBNP, and echocardiographic markers of diastolic function were compared using the independent samples t-test or the Mann–Whitney U test. Linear regression analyses were used to examine the effect of iron deficiency on outcomes independent of other differences in baseline characteristics. A *p*-value of <0.05 was considered statistically significant for all of the analyses. All statistical analyses were performed with R statistics.

## 3. Results

### 3.1. Patient Characteristics

A total of 503 patients were included during the recruitment period; 217 (43%) were female. The mean age of patients was 78 ± 11 years. The majority of patients (55%) had NYHA class IV dyspnea. Complete echocardiographic data was available for 487 out of 503 patients. A total of 160 (33%) had HFrEF, 270 (55%) had HFpEF, and 57 (12%) had HFmrEF. Given the small proportion of patients with HFmrEF in our cohort, they were not included in further analyses.

Differences between HFrEF and HFpEF patients are detailed in Table 1. Patients with HFpEF were significantly older than patients with HFrEF, with mean age 80 ± 10 and 75 ± 12 years respectively. A significantly higher proportion of patients with HFpEF were female (52%) as compared to those with HFrEF (30%). The length of stay (LOS) was not significantly different between the categories of heart failure, nor was the NYHA class. There was no difference in prevalence of coronary artery disease, diabetes mellitus, obesity, chronic kidney disease (CKD), or chronic obstructive pulmonary disease (COPD) between HFrEF and HFpEF. Patients with HFpEF had a higher prevalence of hypertension and atrial fibrillation or flutter; HFrEF patients were more likely to be smokers or have a history of smoking. 

### 3.2. Biomarker Levels

There was no significant difference between groups with regard to CRP levels, although this trended higher in HFpEF patients. Patients with HFrEF had significantly higher BNP and NT-proBNP than the HFpEF cohort.

### 3.3. Prevalence of Iron Deficiency

Both ferritin and transferrin saturation results were available for 416 patients. Of this, a total of 239 patients had iron deficiency, representing 54% of patients with HFrEF and 56% of patients with HFpEF. There were no statistically significant differences in absolute and functional iron deficiency between HFrEF and HFpEF. Haemoglobin level was significantly lower in patients with HFpEF, at 121 ± 23 g/mL, than those with HFREF, at 129 ± 20 g/mL.

### 3.4. Interaction between Iron Deficiency and HFpEF 

**Figure 1:**
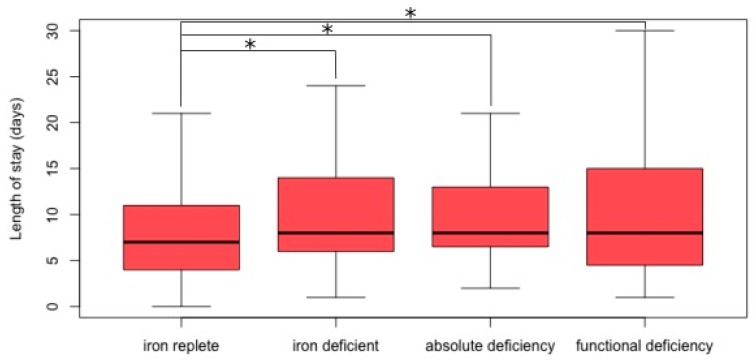
Length of stay according to iron status in heart failure with preserved ejection fraction (HFpEF). Iron replete patients have a significantly lower length of stay than iron deficient patients, across all groups. * denotes *p*-value less than the significance threshold of <0.05.

Table 2 details patients with HFpEF according to iron deficiency status. In patients with HFpEF, females were significantly more likely to be ID than males, across both categories of ID, representing 64% of ID patients, compared to 40% of iron replete patients. There were no significant differences in age or NYHA class determined by ID status. LOS was significantly longer amongst those with ID, irrespective of type (9 days vs. 11 days, *p* = 0.036), as depicted in Figure 1. HFpEF patients with absolute ID were less likely to have CKD than those with functional ID or iron replete patients. Conversely, those with absolute ID were more likely to have COPD compared to iron replete patients. There were no differences across iron status groups in other comorbidities. CRP was significantly higher in those with functional ID, and was lowest in those with absolute ID. BNP and NT-proBNP levels did not differ significantly across ID categories. 

In multivariate linear regression analysis for LOS with iron deficiency as a binary variable, iron deficiency remained an independent predictor of LOS in HFpEF (*b* = 2.74, 95% CI 0.32–5.17), after adjusting for CKD, CAD, hypertension, DM, obesity, atrial fibrillation or flutter, COPD, CRP, and age. The adjusted R2 for this model was 6.5%, indicating significant unmeasured variables accounting for differences in LOS. 

### 3.5. Interaction between Iron Deficiency and HFrEF

Table 3 details patients with HFrEF according to iron deficiency status. In patients with HFrEF, females were not more likely to be ID than males, but there were proportionally more females within the total and absolute ID groups than in the iron replete group, representing 40% of ID patients, compared to 23% of iron replete patients. There were no significant differences in age, NYHA class, LOS, or comorbidities according to ID. Biomarkers CRP, BNP, and NT-proBNP did not differ according to iron status. 

In multivariate linear regression analysis for LOS with iron deficiency as a binary variable along with comorbidities CKD, CAD, hypertension, DM, obesity, atrial fibrillation or flutter, COPD, and age, COPD was the only independent predictor of length of stay (*b* = 42, 95% CI 7.44–76.7). The adjusted R^2^ for this model was 7.6%, indicating significant other active factors accounting for the difference in LOS. 

## 4. Discussion

### 4.1. Prevalence of Iron Deficiency

Our study of ADHF found a high prevalence of ID in both HFrEF and HFpEF, at 54% and 56% respectively. Furthermore, in both patients with HFpEF and HFrEF, women were overrepresented amongst iron deficient patients. Finally, ID was associated with prolongation of hospital stay in HFpEF, but not HFrEF.

With respect to prevalence of ID, these results mirror similar studies of the prevalence of ID in different HF categories. Martens et al. found the prevalence of ID to be 53% and 64% for HFrEF and HFpEF respectively in a cohort of 1197 patients. A higher prevalence of ID was found in a study by Nanas et al., with 73% showing ID on bone marrow aspirate [19]; however these patients were categorized as advanced heart failure, and they only recruited patients with anemia, as compared to our study, which included all patients with ADHF. 

Regarding gender differences in our cohort, with HFpEF women more likely to be ID, this reflects a higher prevalence of ID in women across Europe [20]. Given the link between ID and LOS, along with rehospitalization and functional outcomes in HFpEF in other studies, ID may contribute to the relative over expression of HFpEF in women.

Our findings are concordant with those previously reported in the literature: just over half (55%) of patients had HFpEF [21]; those with HFpEF were more likely to be female and older, with a higher prevalence of hypertension and atrial fibrillation, than those with HFrEF [21]; patients with HFrEF were more likely to be active or past smokers [22]; and natriuretic peptide levels were higher in patients with HFrEF [23].

Our study expands on the limited existing literature regarding the interaction between ID and heart failure outcomes in HFpEF, and notably ID patients with HFpEF had a longer LOS. We found that in the HFpEF cohort, the LOS was approximately 2 days longer in those who were ID; a finding that was not seen in the HFrEF cohort. LOS might be a surrogate finding for a more prominent relationship between ID and HFpEF than HFrEF and this may suggest a prognostic role for ID in HFpEF.

ID affects cardiovascular function through multiple mechanisms. It reduces oxygen carrying capacity and oxygen storage; and in heart and skeletal muscle, it causes mitochondrial dysfunction, reduced energetic efficiency, and anaerobic metabolism [17]. ID is closely linked to exercise intolerance given this role in energy storage and production in both cardiac and skeletal muscle, resulting in reduced work productivity [24]. This mirrors the complex nature of exercise intolerance in HFpEF, which derives from the interaction of pulmonary, cardiac, hematological and skeletal muscle impairments [25]. Given exercise intolerance is a cardinal symptom in HFpEF, this relationship between ID and exercise tolerance may explain the impact of ID on LOS in our study, however data on cardiopulmonary exercise testing or 6-min walk tests would be required to confirm this hypothesis.

ID also plays direct role in cardiac remodeling, as demonstrated by Martens et al. in a recent study showing that cardiac resynchronization therapy (CRT) is less effective at improving left ventricular geometry in those who are ID. Furthermore, ID predicted attenuated symptomatic improvement with CRT [26]. These adverse effects of ID on cellular function and cardiac remodeling were independent of anemia [17,26]. However, remodeling in patients with HFpEF differs from that of HFrEF, and the applicability of these findings to HFpEF patients is not clear. Naito et al. found that long-term ID with anemia in rats resulted in left ventricular hypertrophy with preserved ejection fraction, with associated myocardial interstitial fibrosis [27], suggesting that ID may also play a pathophysiologic role in HFpEF; however, replication of these results in human subjects is required. We did not see any changes to echocardiographic parameters in our HFpEF cohort according to ID category; however, this may have been result of an inadequate sample size. 

A pathophysiological link between ID and HFpEF may arise from their shared inflammatory origins; HFpEF is driven by a pro-inflammatory state secondary to comorbidities [28] as demonstrated by higher levels of circulating inflammatory markers compared to HFrEF [29]; and ID is associated with systemic inflammation which causes elevated levels of hepcidin, leading to functional ID [5]. This was highlighted in our cohort by higher levels of CRP in ID patients with HFpEF. Iron plays a role in regulation of the immune system and response to pathogens [30], however there is no direct evidence to date suggesting that iron deficiency itself causes inflammation. 

The observation of increased LOS in ID patients with HFpEF could be a reflection of mediating comorbidities that cause iron deficiency and that could lengthen the hospital stay. CKD is commonly associated with HFpEF [31], and causes ID through increased hepcidin, bleeding due to uremic platelet dysfunction, impaired dietary iron absorption, and loss through dialysis. Obesity and insulin resistance are associated with altered iron homeostasis, and particularly in females ID is a common feature [32]. We analyzed the contribution of these comorbidities, along with CRP given a possible role of infections or inflammation, and age, in a multivariate linear regression model, in which ID remained an independent predictor of LOS. Whilst the model accounted for only a small portion of the variation in LOS in HFpEF, it remains a contributor to prolonged hospitalization.

Another possible confounder for our finding is that increasing heart failure severity increases the likelihood of iron deficiency [6], and therefore the prolonged LOS in ID patients could be driven by severity of heart failure rather than iron deficiency. However, there was no difference in NYHA class across ID groups in our study, suggesting that this may not explain our finding. 

### 4.2. Clinical Implications

Our study highlights the importance of diagnosing iron deficiency, as it plays a role in determining short-term prognosis. However, iron studies appear to be underperformed in the clinical setting. A study from Silverberg et al. in 2013 found that 72.4% of patients with a primary diagnosis of heart failure had no iron workup [33]. In our study, 82.7% of patients had complete iron studies, representing a significant improvement on the aforementioned study. 

Intravenous iron therapy with ferric carboxymaltose is established as an important treatment for patients with HFrEF, improving functional capacity, symptoms of heart failure, quality of life, and reducing heart failure hospitalizations [34,35]. Given that treatment for HFpEF is limited by a lack of efficacy of neurohormonal therapies [16], and treatment currently focuses on comorbidities and diuresis [18], novel therapeutic strategies are in demand. The effect of ID in prolonging LOS in HFpEF patients in our study may suggest potential efficacy of intravenous iron treatment in this population to reduce hospitalization, and to improve functional outcome. Our findings support the hypothesis of the FAIR-HF trial, which is currently investigating the efficacy of ferric carboxymaltose in the treatment of patients with HFpEF (NCT03074591).

The elevated CRP in functional ID in this study may highlight an alternative therapeutic approach in targeting inflammation in HFpEF. Anti-inflammatory therapies have already proven effective in reducing myocardial infarction, nonfatal stroke, and cardiovascular death [36]. The aforementioned relationship between inflammation, ID, and HFpEF could indicate a role of anti-inflammatory therapies in reducing ID and accordingly improving outcomes in HFpEF patients.

### 4.3. Limitations of the Study

Limitations as well as strengths of our study are its prospective observational design which on the one hand reduces our ability to assess causal relationships, notably because of residual confounding, but on the other hand reduced both selection and recall bias. Further limitations include lack of data on certain parameters of clinical management. For example, data on exercise capacity, such as maximal oxygen consumption testing, was not available. In addition, data on administration of iron infusions was not collected. This limitation may be particularly relevant to patients with HFrEF, who are treated with intravenous iron therapy if ID in accordance with heart failure guidelines [18], unlike patients with HFpEF. This may explain our failure to find any association between ID and LOS in HFrEF, which may have been mitigated by iron infusion treatment, sometimes benefiting patients symptomatically within days of their infusion. Ideally, our study should be replicated with more sophisticated prognostic measures, such as cardiopulmonary exercise testing and invasive hemodynamic measures.

## 5. Conclusions

In this cohort of patients with ADHF, there is a high prevalence of ID in both HFrEF and HFpEF, with a significantly higher proportion of women with ID and HFpEF. Furthermore, ID was associated with prolongation of hospital stay in HFpEF, but not HFrEF. This may indicate an important role for iron deficiency in HFpEF, which could therefore also represent a therapeutic target. Further studies in larger cohorts and more varied populations are warranted to increase the generalizability of these results.

## Figures and Tables

**Table 1:** Patient characteristics, iron status, biomarker levels, and diastolic function indices on transthoracic echocardiography (TTE), according to heart failure category.

	Total Cohort	HFrEF, 160 (33%)	HFpEF, 270 (55%)	*p*-Value
Baseline characteristics				
LVEF (%)	48 ± 16	27 ± 6	60 ± 6	<0.001 *
Age, year	78 ± 11	75 ± 12	80 ± 10	<0.001*
Female sex, *n* (%)	217 (43%)	48 (30%)	141 (52%)	<0.001 *
NYHA class, I/II/III/IV (%)	2/6/37/55	1/7/40/52	1/7/38/54	0.97
Length of stay, days	11 ± 9	11 ± 8	10 ± 8	0.34
Coronary artery disease, *n* (%)	177 (36%)	65 (42%)	83 (32%)	0.054
Hypertension, *n* (%)	393 (79%)	111 (70%)	223 (84%)	<0.001 *
Diabetes mellitus, *n* (%)	157 (31%)	47 (30%)	87 (32%)	0.61
Obesity, *n* (%)	231 (47%)	69 (45%)	130 (49%)	0.49
Active/past smoker, *n* (%)	288 (60%)	106 (68%)	145 (56%)	0.019 *
Chronic kidney disease, *n* (%)	175 (36%)	49 (31%)	105 (40%)	0.081
Atrial fibrillation or flutter, *n* (%)	222 (45%)	58 (36%)	132 (50%)	0.01 *
COPD, *n* (%)	73 (15%)	21 (14%)	38 (16%)	0.61
Iron studies				
Iron deficiency, *n* (%)	239 (57%)	73 (54%)	123 (56%)	0.88
Absolute iron deficiency, *n* (%)	165 (38%)	49 (37%)	82 (41%)	0.64
Functional iron deficiency, *n* (%)	74 (18%)	24 (18%)	30 (15%)	0.61
Haemoglobin level, g/mL	123 ± 23	129 ± 20	121 ± 23	<0.001 *
Biomarker levels				
CRP, mg/L	11.4 ± 27.6	9.4 ± 19.8	12.1 ± 31.5	0.064
BNP, ng/L	857 ± 885	1279 ± 974	648 ± 687	<0.001 *
NT-proBNP, pg/mL	3926 ± 6763	7340 ± 8020	2695 ± 5171	<0.001 *

COPD, chronic obstructive pulmonary disease; LVEF, left ventricular ejection fraction; NYHA, New York Heart Association; BMI, body mass index; LV, left ventricle; LVMI, left ventricular mass index; LAVI, left atrial volume index; RVSP, right ventricular systolic pressure; TTE, transthoracic echocardiography; RVSP, right ventricular systolic pressure; CRP, C-reactive protein, BNP, brain natriuretic peptide, NT-proBNP, N-terminal prohormone of BNP. * denotes *p*-value less than the significance threshold of <0.05.

**Table 2:** Patient characteristics, biomarker levels and diastolic function indices on TTE according to iron status in the HFpEF cohort.

		Iron Deficient			
	Iron Replete	Total	Absolute	Functional	*p*-Value
Female sex, *n* (%)	39 (40%)	79 (64%)	59 (67%)	20 (57%)	0.0012 *
Age	79.1 ± 9.2	79.1 ± 10.2	78.7 ± 10.2	82.8 ± 9.6	0.038 *
NYHA class, I/II/III/IV (%)	2/6/34/44	1/9/45/57	1/3/32/44	0/6/13/13	0.19
Length of stay, days	9 ± 6	11 ± 7.7	11 ± 7	11 ± 9.5	0.036 *
CAD, *n* (%)	36 (38%)	35 (29%)	27 (31%)	8 (24%)	0.30
Hypertension, *n* (%)	84 (87%)	96 (81%)	69 (81%)	27 (82%)	0.58
Diabetes mellitus, *n* (%)	35 (36%)	37 (31%)	26 (30%)	11 (32%)	0.67
Obesity, *n* (%)	46 (47%)	60 (50%)	47 (54%)	13 (38%)	0.28
Active/past smoker, *n* (%)	53 (58%)	71 (59%)	51 (59%)	20 (59%)	0.98
CKD, *n* (%)	50 (53%)	44 (37%)	26 (31%)	18 (53%)	0.008 *
Atrial fibrillation, *n* (%)	50 (52%)	61 (50%)	41 (47%)	20 (59%)	0.50
COPD, *n* (%)	11 (12%)	26 (22%)	22 (27%)	4 (12%)	0.024 *
CRP	13.3 ± 51.6	9.9 ± 26	7.5 ± 20.1	24 ± 48.6	<0.001 *
BNP	768 ± 710	585 ± 463	525 ± 461	1090 ± 236	0.46
NT-proBNP	3239 ± 6814	2389 ± 3174	2318 ± 2294	2515 ± 7058	0.12
EF (%)	60 ± 5	60 ± 7.5	60 ± 7.62	60 ± 7.5	0.97

CAD, coronary artery disease; CKD, chronic kidney disease; COPD, chronic obstructive pulmonary disease; NYHA, New York Heart Association; CRP, C-reactive protein, BNP, brain-natriuretic peptide; NT-proBNP, N-terminal prohormone of BNP; EF, ejection fraction. * denotes *p*-value less than the significance threshold of <0.05.

**Table 3:** Patient characteristics, biomarker levels and diastolic function indices on TTE according to iron status in the heart failure with reduced ejection fraction (HFrEF) cohort.

		Iron Deficient			
	Iron Replete	Total	Absolute	Functional	*p*-Value
Female sex, *n* (%)	14 (23%)	29 (40%)	24 (49%)	5 (22%)	0.007 *
Age, HFpEF	74.7 ± 11.1	76.4 ± 12.4	77.3 ± 13.1	74.5 ± 11	0.32
NYHA class, I/II/III/IV (%)	0/4/23/34	0/6/32/32	0/3/24/20	0/3/8/11	0.74
Length of stay, days	12 ± 8	12 ± 9	11 ± 10	12 ± 6.5	0.78
CAD, *n* (%)	25 (42%)	28 (41%)	19 (40%)	8 (38%)	0.96
Hypertension, *n* (%)	45 (74%)	46 (64%)	32 (65%)	13 (59%)	0.39
Diabetes mellitus, *n* (%)	18 (30%)	20 (28%)	10 (20%)	9 (41%)	0.20
Obesity, *n* (%)	28 (48%)	29 (40%)	16 (33%)	12 (55%)	0.14
Active/past smoker, *n* (%)	37 (64%)	51 (72%)	32 (67%)	18 (82%)	0.30
CKD, *n* (%)	24 (39%)	17 (24%)	10 (21%)	7 (32%)	0.12
Atrial fibrillation, *n* (%)	23 (38%)	24 (33%)	14 (29%)	9 (41%)	0.49
COPD, *n* (%)	9 (15%)	10 (14%)	8 (17%)	2 (9%)	0.74
CRP	11.4 ± 24.6	8.7 ± 19.1	7.9 ± 12.8	11.4 ± 35.6	0.33
BNP	1294 ± 983	1292 ± 1052	1340 ± 678	926 ± 737	0.41
NT-proBNP	7928 ± 7201	6853 ± 10,327	6998 ± 10,787	3938 ± 7196	0.45
EF (%)	27.5 ± 10	27.5 ± 10	27.5 ± 7.5	32.5 ± 8.8	0.083

CAD, coronary artery disease; CKD, chronic kidney disease; COPD, chronic obstructive pulmonary disease; NYHA, New York Heart Association; CRP, C-reactive protein, BNP, brain-natriuretic peptide; NT-proBNP, N-terminal prohormone of BNP; EF, ejection fraction. * denotes *p*-value less than the significance threshold of <0.05.
